# Tropical Forests Are Non-Equilibrium Ecosystems Governed by Interspecific Competition Based on Universal 1/6 Niche Width

**DOI:** 10.1371/journal.pone.0082768

**Published:** 2013-12-30

**Authors:** Hugo Fort, Pablo Inchausti

**Affiliations:** 1 Complex Systems Group, Institute of Physics, Facultad de Ciencias, Universidad de la República, Montevideo, Uruguay; 2 Centro Universitario Regional del Este, Universidad de la República, Maldonado, Uruguay; Universidad Carlos III de Madrid, Spain

## Abstract

Tropical forests are mega-diverse ecosystems that display complex and non-equilibrium dynamics. However, theoretical approaches have largely focused on explaining steady-state behaviour and fitting snapshots of data. Here we show that local and niche interspecific competition can realistically and parsimoniously explain the observed non-equilibrium regime of permanent plots of nine tropical forests, in eight different countries. Our spatially-explicit model, besides predicting with accuracy the main biodiversity metrics for these plots, can also reproduce their dynamics. A central finding is that tropical tree species have a universal niche width of approximately 1/6 of the niche axis that echoes the observed widespread convergence in their functional traits enabling them to exploit similar resources and to coexist despite of having large niche overlap. This niche width yields an average ratio of 0.25 between interspecific and intraspecific competition that corresponds to an intermediate value between the extreme claims of the neutral model and the classical niche-based model of community assembly (where interspecific competition is dominant). In addition, our model can explain and yield observed spatial patterns that classical niche-based and neutral theories cannot.

## Introduction

The classical competition niche theory (CCNT) based on the Lotka-Volterra competition equations and Hutchinson's multidimensional niche [1], [2] generally predicts the coexistence of a relatively large number of species with disjoint niches at equilibrium. At the expense of substantially increasing their complexity (as measured by the number of parameters), more complex versions of these classical models include variation in the values of model parameters across space (for instance in different micro-habitats or patches) and differential species dispersal between different sub-habitats and may predict the equilibrium coexistence of a larger number of species [3]. Thus, neither model may account for and thus explain the non-equilibrium coexistence of hundreds of species in megadiverse communities such as saturated tropical forests [4]. More recently, the neutral theory of biodiversity (NTB) [4] assuming that ecological dynamics stem from the ecological drift of functionally equivalent species with identical niches, has been able to predict the relative species abundances (RSA), the species-area relationship (SAR) and main biodiversity indices in tropical forests with only four parameters with an accuracy and consistency that have eluded the classical niche-based theory [4]–[5]. In the same vein as the classical niche-based theories, the NTB also predicts the (stochastic) equilibrium of community metrics with the species loss due to ecological drift being balanced by dispersal and/or speciation [4]. The NTB has a clear gain in predictability of macroscopic (RSA, SAR, etc) variables with a few parameters compared with the classic niche-based approaches [4]–[5]. However, not being a spatially-explicit theory, the original, standard NTB cannot predict distinctive features of the spatial dynamics of megadiverse tropical forests [6]. The NTB has also been criticized for ignoring the strong evidence of functional [7] and fitness differences [8] among tree species and for incorrectly predicting the observed rates of species turnover [9] and of spatial differentiation at biogeographic scale [10].

We believe that CCNT and NTB share two fundamental structural limitations when applied to understand the dynamics of tropical forests. First, they rely on equilibrium solutions to understand the community dynamics of these forests and their theoretical predictions are typically compared with census data of tropical forest plots viewed as snapshots [4], [11]. However, these large, permanent tropical forests plots that have been exhaustively and repeatedly censused over time [12] clearly reveal that tropical forests are far from stationary. For example, the plot at Barro Colorado Island (Panama) has lost 37 species in 23 years, and the plot at Bukit Timah (Singapore) had an average rate of species loss of 8% between consecutive censuses [11]. Being far from equilibrium, fitting detailed snapshots of tropical forest data can only describe transitory configurations but cannot help to understand the mechanisms underlying the observed dynamics. The second problem of both CCNT and NTB is the reliance on mean-field approximations stemming from the “well-mixed” assumption that clearly fails when applied to sessile individuals like trees having largely local recruitment and potentially suffering from local interference competition for resources. Thus, neither CCNT nor NTB are suitable for the realistic, parsimonious modelling of tropical forest dynamics, nor can they predict the large array of metrics characterizing the spatio-temporal dynamics of these forest communities.

Here we propose what in our view is the simplest non-equilibrium, spatially explicit model of community dynamics of tropical forests. In this model, trees of different species compete locally with neighbouring individuals depending on their degree of niche overlap. This model does not pretend to generate “photorealistic” representations of tropical forest plots (i.e. to build a model depicting the actual locations occupied by each tree species and reproduce their dynamics). Rather our aim is to formulate a minimal set of realistic biological features sufficient to accurately predict different commonly used biodiversity metrics as well as their evolution. In this regard, we chose the simplest possible niche space i.e. a finite, one-dimensional axis with all species having identical niche width and, as in NTB [4], our model did not consider the size structure of populations. This is not to imply that ecological niches are homogeneous and one-dimensional and that species' size structures are irrelevant to understand the dynamics of tropical forests, but that adding further complexity to the model was unwarranted at this stage. Competitive interactions among individuals of sessile, seed-bearing plants are known to take place within limited neighbourhoods with locally changing species composition [13]. This, together with the strong dispersal and recruitment limitation of tropical tree species [14], required formulating a spatially explicit, individual-based model [15]. Always maintaining the maximum possible simplicity, we represented the positions of individual trees in a regular square lattice i.e. a cellular automaton (CA) model [16]. Therefore, interspecific competition occurs if and only if individuals of different species are both neighbours in space and the species are relatively close along the niche axis so as to have important niche overlap that determines the strength of their competitive interaction (see Methods). While other individual-based e.g. [17]–[19] and mean-field e.g. [20]–[21] models of community dynamics have been proposed, their focus had been the contrast between modelling predictions of niche-based and neutral theories rather than the validation of theoretical predictions through the comparison with empirical data.

## Results and Discussion

**Figure 1:**
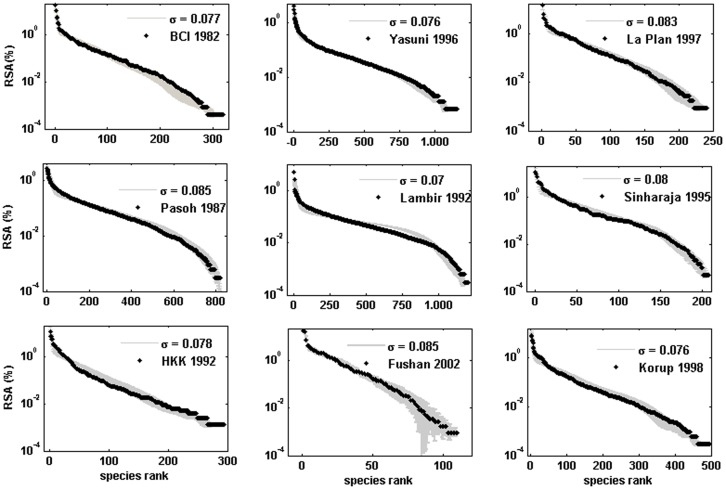
Observed (bold) and predicted distribution of relative species abundances (RSA) for all trees with diameter at breast height (dbh)≥1 cm for the first census of nine in nine tropical forest plots. Data from Center for Tropical Forest Science [11]. The predicted (grey) are averages ± std of 100 model simulations for the best estimates of model parameters of each forest (see Information S1 for comparisons with other censuses). The calculation of the RSA is explained in Information S1.

**Figure 2:**
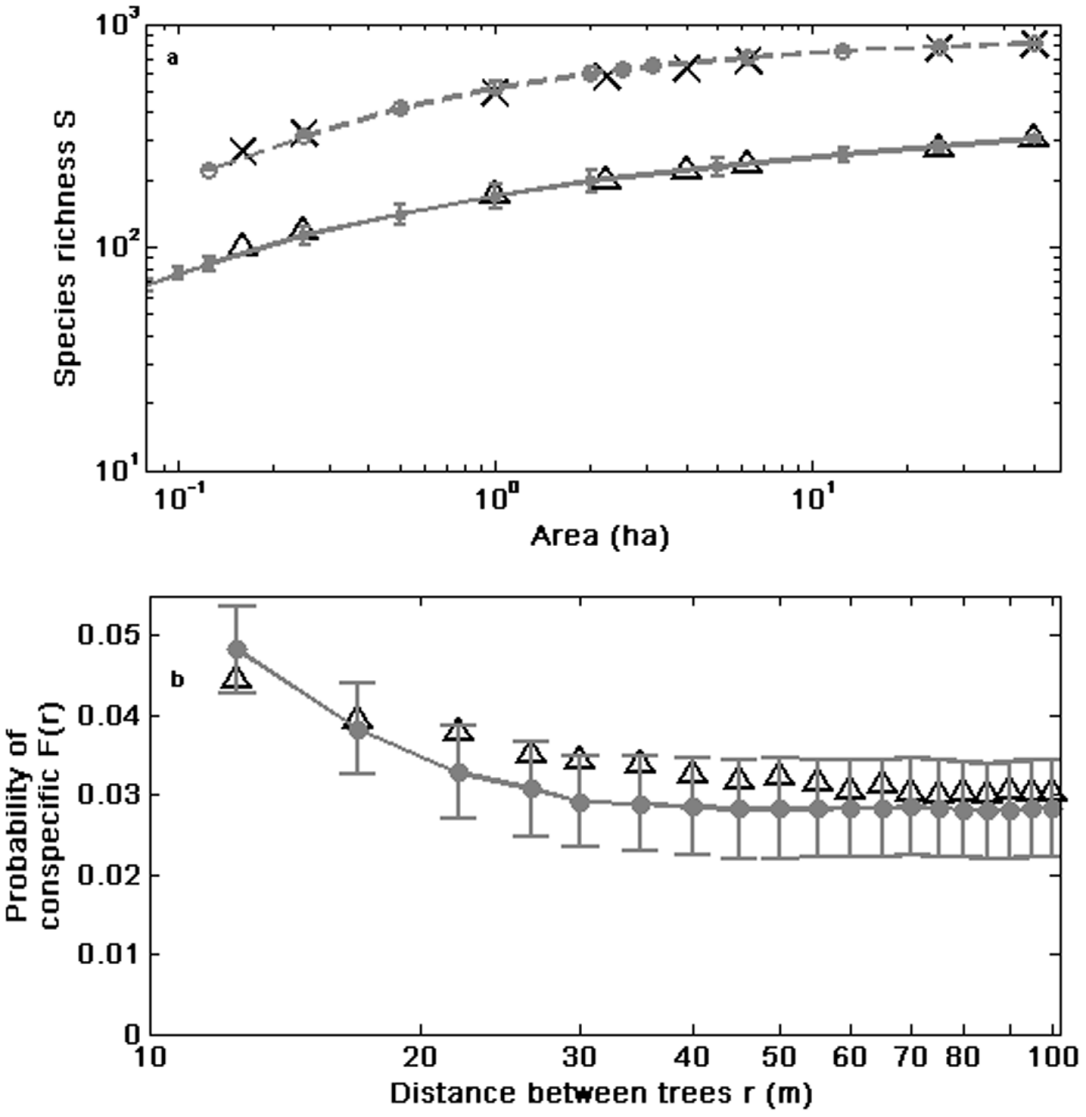
Species-area curves (SAR) and spatial patterns of tree species richness for selected censuses of tropical forests. Predicted curves correspond to averages over 100 simulations for the best estimates of model parameters (Fig. 2a), and the error bars correspond to one std. **a.** Observed and predicted (grey line) number of tree species with dbh≥1 cm for sampling areas of different sizes at Barro Colorado (1990, triangles) and Pasoh (1987, crosses). Estimated curves for Barro Colorado and Pasoh were calculated using data from the Center for Tropical Forest Science [11] and dividing the entire plots into non-overlapping quadrats [29]. The calculation of the SAR is explained in Information S1. **b.** The estimated (triangles) and predicted probability *F*(*r*) that two randomly selected trees of dbh≥10 cm located *r* meters apart for the 1990 census at Barro Colorado plot are conspecific. The curves are shown only for 10≤*r*≤100 m, a range of distances for which the NTB fails to reproduce the estimated *F*(*r*) [6].

**Figure 3:**
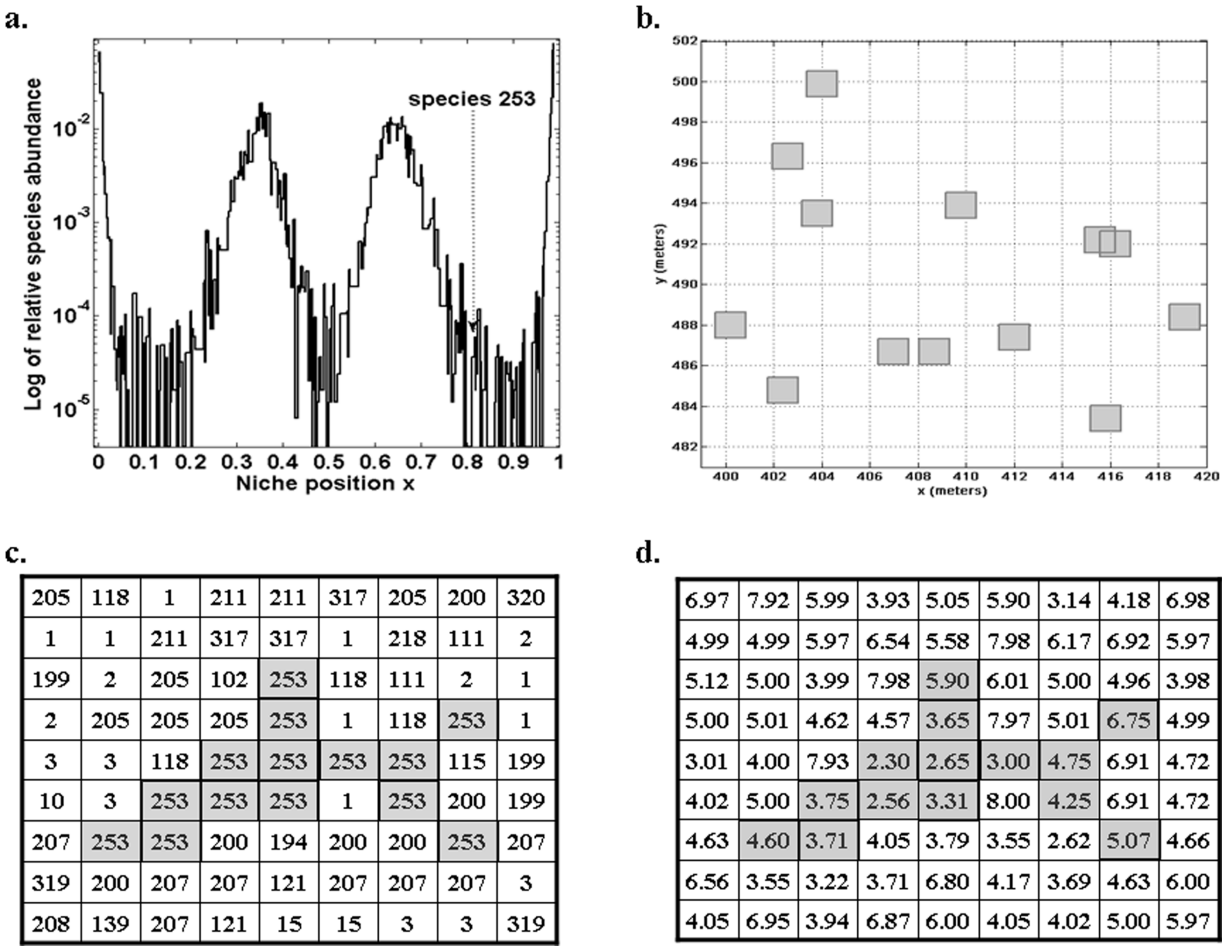
Relation between species rarity and spatial aggregation for *Spachea membranacea* at Barro Colorado in the 1995 and model species # 253. Both species have the highest spatial aggregation, as measured by the aggregation index Ω_0→10_ and the same abundance of 14 individuals. **a.** The clumpy distribution of relative abundance of species in the finite niche axis, showing that species # 253 (niche position *x* = 0.8179) lies in a gap between clumps of coexisting species. **b.** The observed distribution of *Spachea membranacea* at Barro Colorado in the 1995 census [11], [24] yielding Ω_0→10_ = 689.3. **c.** Spatial distribution of a model species # 253 in grey. The selected 9×9 sublattice shows the species identity of individuals in the immediate neighbourhood containing all 14 individuals of model species # 253, yielding Ω_0→10_ = 1031.7. **d.** The set of fitnesses in the same sublattice containing all 14 individuals of model species # 253. Individuals of the rare species # 253 (in grey) are poorer competitors because they have lower fitnesses than most of their immediate neighbours.

**Figure 4:**
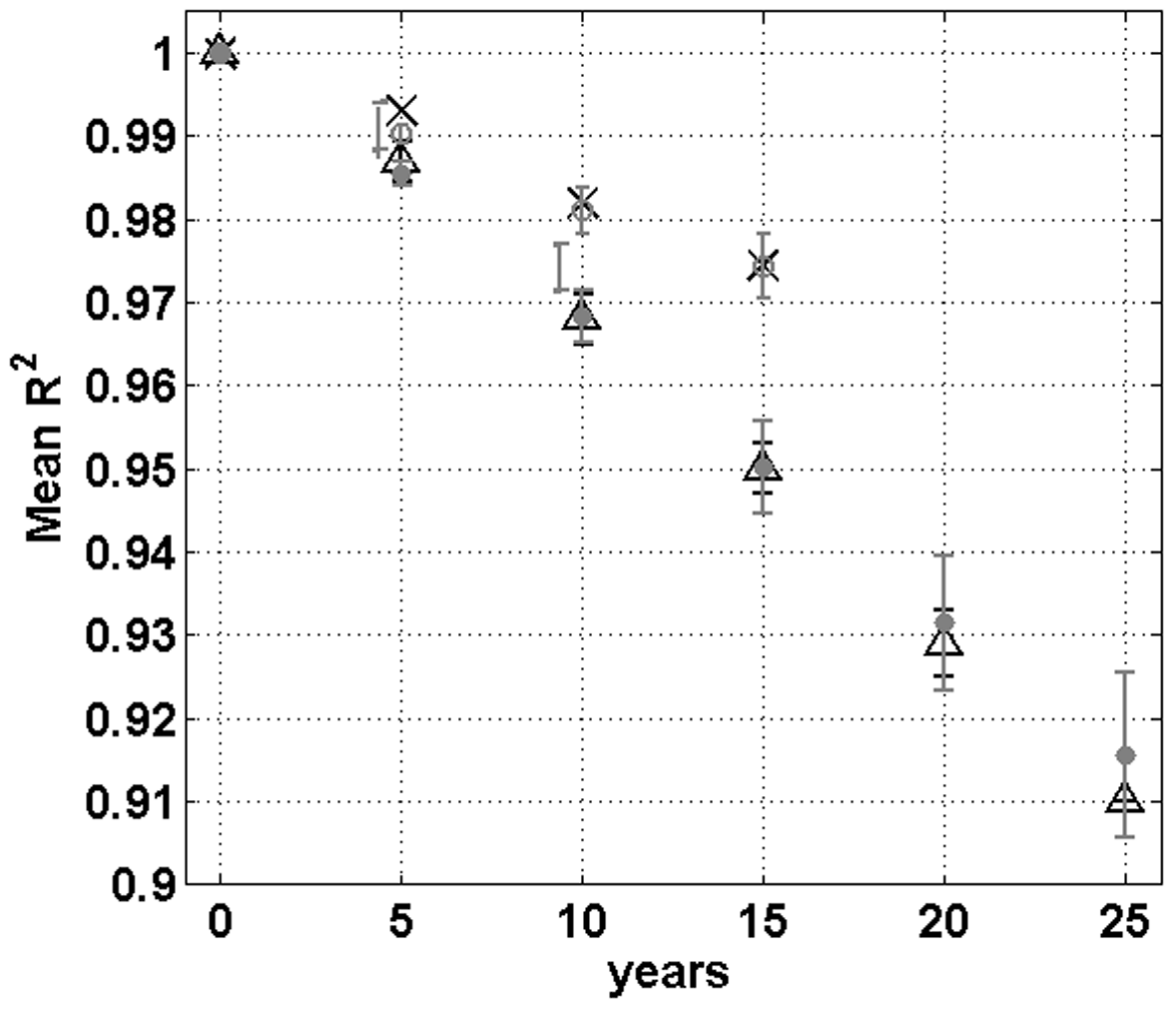
Observed and predicted compositional changes in tree communities between censuses at Pasoh and Barro Colorado forests. Compositional changes are measured by the coefficient of determination *R* ^2^ of the regression of the log-transformed, time-lagged population abundance of all species between censuses for all individuals with dbh≥1 cm and species with two or more individuals in first census of each forest [25], [26]. At a time lag of zero, no change in community composition can yet have occurred, and thus *R* ^2^ is by definition equal to unity. As time elapses between censuses, the progressive compositional changes are reflected by the decay in *R* ^2^: empirical results for Barro Colorado (triangles) and Pasoh (crosses), and predicted values, corresponding to averages over 100 simulations with error bars equal to one standard deviation for the best parameters estimates for each forest. The model predicts a nearly perfectly linear decay in average values of *R* ^2^ that is indistinguishable from the prediction from NTB.

The model was able to fit the main biodiversity metrics used to characterise community structure such as RSA for the nine tropical forest plots studied (Table 1, Fig. 1) and SAR for the two plots for which this information was available: Barro Colorado and Pasoh (Fig. 2a) with similar accuracy as NTB. Furthermore, the model could also fit and explain other biodiversity metrics related to spatial aggregation that NTB cannot. First, the model accurately predicted the short-scale (<100 m) probability *F*(*r*) that two randomly selected trees *r* meters apart inside a plot are conspecific (Fig. 2b). The NTB can not accurately predict the rate of decay of *F*(*r*) at short distances [22]. The local nature of competitive interactions and dispersal limitation (largely within a neighbourhood) can account for the behaviour of *F*(*r*) at small distances (Fig. S1 in Information S1). This fast-decay of the spatial correlations between sites is quite common in many cellular automata with short range interactions [16]. Second, we also obtained the observed tendency of rarer species to be more strongly aggregated in tropical forests, a feature that remains to be explained [6]. Our results suggest that this pattern can be understood in terms of local competition and is related to another finding obtained from our model: the RSA distribution represented on the niche axis displays a pattern with clumps and gaps (Fig. 3a) that is qualitatively similar to the one obtained using Lotka-Volterra competition equations [23]. We found that the niches of rarer species were located in the gaps between clumps in the niche space and that these rare species turned out to be poorer competitors. This association can be illustrated by considering the species with abundances that exhibited the highest values for the aggregation index Ω_0→10_ for both the empirical and theoretical cases. For instance, *Spachea membranacea* had an abundance of 14 trees and Ω_0→10_ = 698.3 in Barro Colorado in the 1995 census [24]. The corresponding theoretical species # 253 for a particular simulation of Barro Colorado in 1995 also had an abundance of 14 individuals and Ω_0→10_ = 1031.7 (Fig. 3c). Notice from Fig. 3d that the fitnesses *f* of individuals of species # 253 were in general low. The association between rarity and spatial aggregation arose because a rare species in our model could only avoid competitive displacement by being surrounded by either conspecifics or by individuals of other species with which they had a minimal niche overlap. Nevertheless, we want to remark that any similarity between the actual spatial distribution of individuals of *Spachea membranacea* and of the theoretical species # 253 shown in Figs. 4b and 4c is pure coincidence.

**Table 1:** Observed (bold) and predicted species richness for all trees with diameter at breast height (dbh)≥1 cm for the first census in nine tropical forest plots.

Forest	Area (ha)	*H*_1_	*L*	*σ, m, T*	*Species richness, S_1_*
Lambir	**52**	**0.863**	600	0.073, 0.05, 0.2	**1204, 1159**
(Malaysia)					1204, 1160±9
Pasoh	**50**	**0.842**	580	0.085, 0.11, 0.5	**823, 819, 811, 808**
(Malaysia)					823, 821±2, 815±4, 808±5
Yasuni	**50**	**0.830**	390	0.076, 0.13, 2.0	**1154, 1087**
(Ecuador)					1154, 1091±8
La Planada	**25**	**0.745**	340	0.083, 0.09, 1.0	**241 221**
(Colombia)					241, 222±7
Sinharaja	**25**	**0.741**	450	0.076, 0.08, 0.0	**207, 205**
(Sri Lanka)					207, 205±2
Korup	**50**	**0.718**	570	0.076, 0.13, 0.3	**495**
(Cameroon)					495
Barro Colorado	**50**	**0.694**	500	0.077, 0.10, 3.0	**320, 318, 303, 299, 292, 283**
(Panamá)					320, 314±4, 300±5, 293±6, 287±7, 281±7
Fushan	**25**	**0.692**	340	0.085, 0.09, 0.0	**110**
(Taiwan)					110
Huai Kha Kaengh	**50**	**0.682**	280	0.077, 0.12, 0.0	**295**
(Thailand)					295

Data from Center for Tropical Forest Science [11]. The predicted values of species richness correspond to averages ± std of 100 model simulations for the best estimates of model parameters. *H*_1_ is the equitability or standardized Shannon-Weaver diversity for the first census in each forest, *L* is the lattice size and the parameter values providing the best fit to empirical data where *σ* is the species' niche width, *m* is the dispersal rate from outside each neighbourhood and *T is* the stochasticity parameter.

A central finding was that the species niche width *σ* in all the nine tropical forests analysed was roughly 0.08 (0.078±0.005) and hence that 95% of each species' niche corresponded to roughly 1/6 of the entire niche axis. It is quite remarkable that only a narrow set of niche widths allowed fitting the spatial and temporal dynamics in all tropical forests considered. This seemingly universal feature echoes the widespread convergence of functional traits leading to shade tolerance and nutritional and hydric niches axes leading to a large niche overlap in tropical tree species [14]. The fact that 2*σ*≈1/6 implies by eq. (2) that the strength of interspecific interactions is on average one-quarter of the intraspecific competition which, by construction, is equal to unity i.e. 〈*α_ij_*〉≈0.25〈*α_ii_*〉. The ratio (〈*α_ij_*〉/〈*α_ii_*〉)≈0.25 corresponds to an intermediate value between the extreme claims of the neutral model (where species are functionally identical and have independent dynamics) and the classical niche-based model of community assembly (where interspecific competition is dominant). There is strong evidence of density dependent regulation through intraspecific competition acting over short distances in tropical trees species [14], [25]. The strength of interspecific competition predicted by our model was however closer to neutrality than to classical niche-based theories of interspecific competition [4].

A unique feature of our model is its capacity to accurately predict the observed dynamics of the species richness (Table 1) and of the RSA distributions (Fig. 1; Figs. S2–S4 in Information S1) in the six permanent plots censused more than once (Lambir, Pasoh, Yasuni, La Planada, Sinharaja and Barro Colorado; Fig. 1). Most analyses of tropical forests to date have considered and predicted biodiversity metrics of consecutive censuses as independent, isolated snapshots [4], [12]. To our knowledge, this is the first mechanistic model capable of realistically and parsimoniously explain the observed dynamics of the main biodiversity metrics used to characterise community structure in tropical forests. In addition, for the two permanent plots for which there were at least four censuses (Pasoh and Barro Colorado), the model could predict the observed compositional changes of tree communities over time (Fig. 4) using the decay in the coefficient of determination *R* ^2^ [26]–[27]. Furthermore, the observed decay in community similarity for these forests was linear and consistent with the very slow convergence to distant equilibrium in community composition [26] that our model would yield if ran for a exceedingly long time frame (approximately 10^9^ individual replacements). This equilibrium state would consist in the stable coexistence of only a handful tree species after all others drifted away to extinction, which is exactly the result found by NTB in the absence of speciation countering species loss [4]. Had we been interested in applying the model for much longer time frames, we would have needed to consider speciation but the latter could be safely ignored when predicting community dynamics at the time scale of years to decades.

### Conclusions

Much like in statistical physics, our model shows that it is unnecessary to model the detailed “microscopic” dynamics in a landscape to accurately predict the aggregate, macroscopic variables characterising the composition and dynamics of large and complex ecosystems such as tropical forests. In fact, we found that local competitive interactions coupled with limited, stochastic dispersal can give rise to the non-equilibrium dynamics for a seemingly universal range of niche widths identical to all tree species that may be interpreted as “neutral”. The latter may explain why many results reported here could be indistinguishable from those predicted by the NTB. Other modelling results have shown that neutral-like patterns of community structure need not imply that neutral processes drive community dynamics [28]. However, the functional similarity among species than can be interpreted as neutrality is not a fundamental building assumption of our model but rather the emergent outcome [23] arising from the interplay between slow competitive displacement among functionally equivalent species and dispersal limitation, both of which are known to occur in tropical forests [26]–[28].

Large plots of tropical forests exhibit a noticeable decay of tree species richness over the scale of years to decades [4]–[5], [24] thus requiring to treat them as non-equilibrium communities. While complex systems at equilibrium can be fully described by their most likely statistical configuration as the NTB [4] and the Maxent theory [29] do, describing non-equilibrium systems requires including the mechanisms that bring about their dynamics [30], namely local competitive interactions and limited dispersal among functionally equivalent species. It is during the long transient, out-of-equilibrium regime that our model predicts the species niche widths seemingly have a universal value of 2*σ*≈1/6 in all forest plots. Further, our model predicts and explains the set of metrics describing the non-equilibrium dynamics of community structure and the spatial patterns of species distribution while others have successfully fitted different community metrics such as RSA [4], [12] and SAR [31]–[32] only after assuming equilibrium. Having a complexity comparable to the NTB, our mechanistic model may constitute an alternative to high-dimensional niche-based models of interspecific interactions as well as to provide further insights on the spatio-temporal community dynamics of tropical forests.

## Methods

### The Model

All *L×L* cells of the CA are occupied by one individual, representing a tree belonging to a given species *s* = 1, 2,…,*n* (i.e. the number of individuals, *N* = *L×L*, remains constant as in the NTB [4]). The entire community was closed to dispersal from the outside and we consider periodic boundary conditions to avoid border effects. We assumed the simplest one-dimensional, finite niche scaled in the unit interval wherein the resource utilization function of each species *s* is defined by a normal distribution *P*(*s*) whose mean *μ*(*s*) and standard deviation *σ*(*s*) indicate the position and width of its niche. The positions of the species niches were chosen by randomly drawing the values of *μ*(s) from a uniform distribution at the beginning of each simulation and were not changed during the simulation. Each focal individual, located at site *i*, belonging to a species *s_i_* only interacts with its eight immediate neighbours (that together with *i* define the Moore neighbourhood *M_i_*). This is of course a simplification, since the effective neighbourhood size of tropical trees on average involves a larger numbers of neighbours [22]–[23] but it varies significantly with the focal species [33]. The strength of its competition with a neighbour of species *s_j_* located at site *j*, *α_ij_*, is proportional to the niche overlap between species *s_i_* and *s_j_*, and can be written as [2] (see also [34] and references therein):
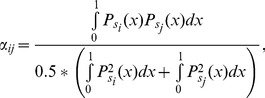
(1)which in turn can be expressed in terms of the standard *error function* “erf” as:
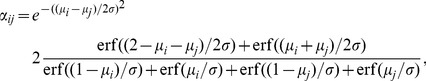
(2)where *μ_k_*≡*
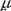
* *s_k_*) and *σ_k_*≡*
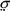
s_k_*). We further assumed that all species were functionally and demographically equivalent by having the same niche width: *σ_i_* = *
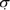
* (which could be regarded as an average niche width). Hence, the fitness *f(s_i_)* of a focal individual of species *s_i_* located at site *i* is given by 
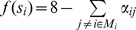
, where the “8” corresponds to the numbers of neighbours of *i* and it ensures that *f(s_i_)* is always non-negative. Thus, *f(s*_i_) has its maximum value when the focal species *s_i_* has minimal overlap with its eight neighbours, and minimal when their niche overlaps are maximal. The functional equivalence between species is consistent with the chosen normalization for the *α_j_* (Eq. 1) to assure that the matrix *α* is symmetric.

Model dynamics consisted on the sequence of individual replacements by another individual (of the same or other species) in each spatial location. Individual replacements are modelled as a stochastic CA [35]–[36]. Hence, each simulation step consists of the potential replacement of one of the *L×L* individuals. The model contains three parameters that dictate the dynamics of individual replacements: the width of each species niche *α*, the dispersal rate from outside each neighbourhood *m*, and the stochasticity parameter *T*. The replacement rule was as follows: (I) A focal individual of species *s_i_*, located at site *i*, is randomly chosen (with probability 1/*L×L*). (II) This focal individual is replaced with probability *m* by the descendant of another randomly chosen individual of species *s_k_* from outside its neighbourhood *M_i_*. And with probability (1-*m*)*Pr(s; *s_i_*→*s_j_*) by a randomly chosen neighbour of species *s_j_* where Pr(s; *s_i_*→*s_j_*) is given by the Glauber update rule 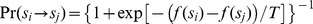, commonly used in lattice spin models [37]–[38]. Thus, the probability that the focal individual *s_i_* was replaced by its neighbour *s_j_* was greater as the difference between individual fitnesses *f*(*s_j_*) and *f*(*s_i_*) increased. The parameter *T* modulated the probability of deterministic replacement to the difference in fitnesses between each pair of species *s_i_* and *s_j_*. The larger the value of *T*, the closer Pr(*s*_i_→*s_j_*) approaches to ½ i.e. the replacement of the focal individual by a neighbour is a totally random process. On the contrary, as *T* approaches its minimum value of 0, Pr(*s*_i_→*s_j_*) approaches to 1 and the change of the focal individual from *s_i_* by a local neighbour *s_j_* becomes deterministic and is accepted if and only if *f*(*s_i_*)<*f*(*s_j_*). Therefore, both *m* and *T* introduce stochastic variation in the local replacement of focal individuals in the model: the former as the recruitment from dispersal of any species outside each local neighbourhood through the seed dispersal by wind or animals and the latter in the identity of the species replacing the focal individual. Our modelling of local recruitment reflects the well-known dispersal limitation observed in tropical forests whereby most recruits are found within a few meters of the focal individual that produced them [14], [25].

For each forest, we simulated the dynamics for different initial conditions involving each time a random choice of the species niche positions {*μ*(*s*)} (drawn from a uniform distribution), and of the spatial positions for all L individuals (in such a way that each species *s* had the same probability (1/*L×L*) of occupying each site).

### Estimation of Parameters

Our model was tailored to explain the spatio-temporal dynamics of all trees of diameter at breast height (dbh) ≥1 cm in nine large (>25 ha), permanent plots of tropical forests in eight different countries that constitute the best available data of these mega-diverse ecosystems. These permanent tropical forest plots are essentially saturated, *i.e.* the total number of individuals increases linearly with the area inventoried [4], [14]. Therefore, for each analyzed plot, *L* was chosen in such a way that *L×L* was the closest multiple of ten to the maximum number of trees (with dbh≥1 cm) measured along the different censuses, 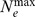, while the initial number of species, *n*, was set equal to the value of the species richness found for the first census, 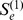 (the subscript *e* stands for “empirical” and then 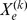 will denote the observed value for quantity *X* in census number *k*). For example, for Barro Colorado (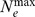 = 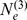 = 244,080 for the third census of 1990 and 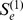 = 320 for 1982): *L* = 500 and *n* = 320.

**Figure 5:**
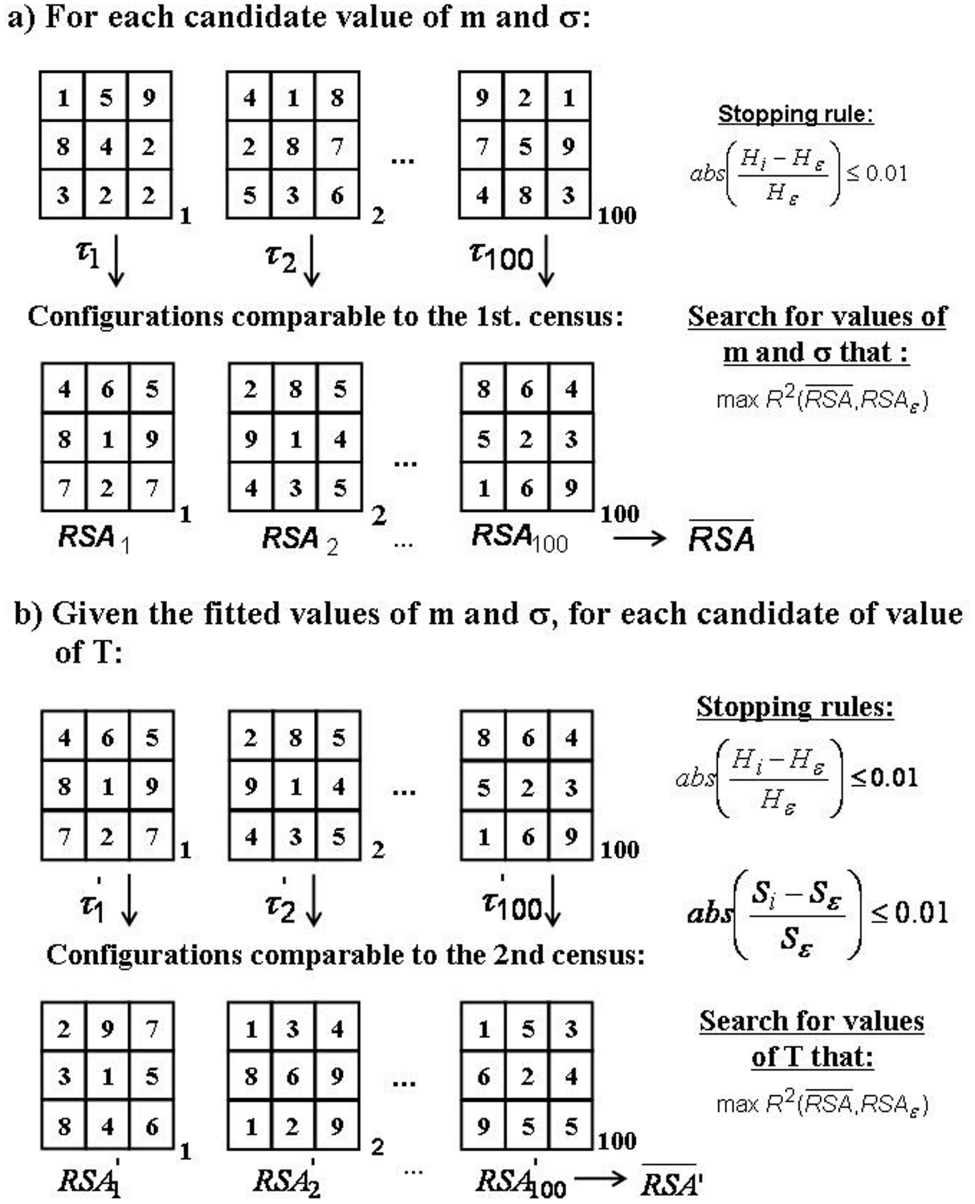
Sequential procedure used to estimate the model parameters for each forest illustrated with a hypothetical grid of 3×3 whose nodes are occupied by different species. a. In the first stage, forest dynamics starting from 100 random initial conditions (central niche positions *μ*(*s*) for the s species and spatial positions for all L individuals dynamics, both drawn from uniform distributions), dynamics consisted on the sequence of τ_i_ (i: 1…100) individual replacements (see rules in the main text) until reaching a configuration whose standardised Shannon diversity was similar to the one observed for the first census. We constrained the species richness to be equal to the one observed in the first census. We then searched for the values of m and σ yielding the maximum value of the coefficient of determination R^2^ obtained by regressing the relative species abundance (RSA, averaged across the 100 simulations) distribution on the RSA of the first census (provided that R^2^≥0.95). **b.** For the fitted values of m and σ, the second stage estimated the value of T for those forests having more than one complete census. Starting for the 100 configurations comparable to the first census, we restarted the sequence of τ′_i_ (i: 1…100) individual replacements (now letting species to become extinct) until each configuration had a standardised Shannon diversity or species richness (whichever came first) similar to the one observed for the second census. As in the first step, we searched for the value of T yielding an average RSA similar to the one observed in the subsequent censuses, as judged by the R^2^ criterion as in step (a).

It is unfeasible to estimate the values of parameters *m*, *σ*, and *T* using maximum likelihood methods and the data from the permanent forest plots because the potential number of local replacement rules quickly becomes cumbersome as the number of states (species) and neighbours increase. In our case, the number of local replacement rules would be 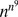 the “9” comes from the size of the Moore neighbourhood. We used an alternative method based on a sequential procedure to estimate the parameters *σ*, *m*, and *T* providing the best fit to the observed dynamics as characterised by a set of common metrics in the set of censuses of each forest (Fig. 1). Actually, it turns out that while *σ*and *m* were enough to reproduce with accuracy the values of all biodiversity metrics found at the first census of each forest plot, *T* was a “fine tuning parameter” required to improve the agreement between observed and predicted values of the RSA and the Shannon equitability index *H* = 
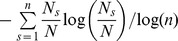
 (where *N_s_* is the abundance of species *s*) calculated for subsequent censuses. For this reason *T* = 0 for all the forest plots for which there is only one census (see Fig. 1a). Thus in a first stage (Fig. 5), using only data of the first census, we estimated *σ* and *m*. To do this we systematically searched the array of values in the plane *σ* -*m* generated by varying *σ* in (0.05, 0.1) in steps of Δσ = 0.001 and *m* in (0.02, 0.12) in steps of Δ*m* = 0.01. However, given that forests are non equilibrium systems, it is unknowable how many simulation steps are required to yield a configuration comparable to the one observed in the first census starting from different initial conditions. For each given pair (*σ*, *m*), we generated 100 initial conditions (see above) and ran each simulation until the predicted value of *H* was equal to the empirical 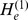 for the first census with an accuracy of 1%. This comparison allowed deciding when to stop each simulation. We prevented individual replacements of species having only one individual in order to constrain the CA configuration corresponding to the first census to the observed 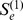 species. For CA configuration stopped when the predicted H was sufficiently close to the empirical value, we chose the pair of values of *σ* and *m* such that the coefficient of determination 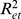 of the linear regression between the observed and predicted (average over the 100 simulations) RSA distributions, was the highest provided that 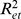
≥0.95.

In a second stage (Fig. 5), the pair of fitted values of *σ* and *m* was then used to estimate the other parameter, *T*. We restarted the simulation at each CA configuration corresponding to the first census now allowing species to become extinct so that the predicted forest dynamics could describe the observed changes in *S* for the remaining censuses of each forest. Proceeding in a similar way as before, we systematically searched for the best fitting value of *T* in (0.5, 5.0) in steps of Δ*T* = 0.5. For each candidate value of *T*, the number of simulation steps between consecutive censuses for a given simulation was set whenever the absolute values of (*H*−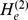)/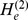 or (*S*−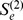)/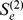 became ≤0.01 (the first that was satisfied). As before, the best estimate of *T* was the one that predicted the highest 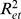 for all censuses provided that it was greater than 0.95.

### Computation of biodiversity metrics

We estimated the average values of the metrics commonly used to characterise the spatial distributions of tree populations and the structure and dynamics of tree communities for the set of censuses of each forest, and used them to compare predicted and observed forest dynamics. These metrics were: the RSA, the species richness, the Shannon equitability, the SAR, two indices of population aggregation (Ω_0→10_ and *F*(*r*)) and the similarity in community composition over time. In all the cases except the aggregation *F*(*r*) we focused on trees with dbh≥1 cm.

The RSA was calculated by counting the number of species falling in each ranked abundance interval [4]. The SAR (average number of species vs. plot area) curves were calculated by dividing the entire plot into non-overlapping quadrats of square and rectangular shapes and the number of species present in each counted [39]. We considered quadrats of the following sizes: 5×5, 5×10, 10×10, 10×20, 20×25, 25×25, 50×25, 50×50, 100×50, 100×100, 250×100, 250×250 and 500×500, containing from 25 to 250000 individuals because all sites are occupied in the model The mean number of species in quadrats of different sizes yielded the SAR.

The population aggregation index Ω_0→10_ for each species corresponded to the density of neighbouring conspecifics on quadrats of radius *r* (squares of side 2*r*+1 lattice spacing) relative to the overall density [6]. The value of *r* is such that it corresponds to 10 m for the plot; e.g. for Barro Colorado 500×500 = 250,000 cells correspond to 500,000 m^2^, then the lattice spacing corresponds to 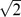 m and r≈7. Aggregation is indicated when Ω_0→10_>1, and overdispersion when Ω_0→10_<1. The probability *F*(*r*) of finding a conspecific at distance *r* when randomly sampling two trees was calculated for Barro Colorado for all individuals with dbh≥10 cm in 1990 [40]. Therefore, in order to compute a comparable aggregation index, we had to shorten the lattice from *L* = 500 to *L* = 150 because there were 21,237 trees with dbh≥10 cm rather than 244,062 trees with dbh≥1 cm .

Modelling a non-equilibrium dynamics requires specifying when the predicted dynamics reflected in the set of computed metrics will be compared with the actual data from the permanent plots. Unlike other modelling approaches focusing on the temporal evolution of states variables (e.g. species individual abundances) that could change values on a predefined time scale, our modelling framework focused on potential replacement events of randomly chosen individuals per simulation step. In each simulation, the sequence of replacement events led to different configurations of species occupancy and to a different set of values of the biodiversity metrics. Consequently, the predicted values of the biodiversity metrics matched the observed values in the forest censuses after different numbers simulation steps (and different numbers of individual replacement events) in each of the 100 simulations. We found that the average number of simulation steps necessary to predict values of the biodiversity metrics similar to the observed ones was directly proportional to the total number of trees *N* (the larger the number of trees, the larger the replacement attempts needed) and to the number of species that went extinct between consecutive censuses Δ*S* (idem), and inversely proportional to the singletons *Σ*_1_ (which represents the species most likely to become extinct). Overall, we found that the number of simulation steps required to fit the data between two consecutive censuses was approximately equal to 7.2×*N*×Δ*S*/*Σ*_1_ for the six forest plots that were censused more than once. We stress that the number of simulation steps required to fit the data between two consecutive censuses does not correspond to the actual replacement events occurring between consecutive censuses in each forest. One reason is that by assuming a neighbourhood for competitive interactions smaller than the ones found for tropical trees [22], [32], the spatial propagation of the replacements occurred slowly in the model. As a consequence, the model required a larger number of replacement events to match the values of observed biodiversity metrics than would have been required if we had chosen a larger sized neighborhood. Finally, the similarity in community composition over time was estimated by the decay in the coefficient of determination *R* ^2^ of the log-transformed abundances of each species between censuses provides a simple measure of community change over time [25], [26]. We calculated R^2^ only using data for species whose abundance was greater than two individuals in the first census of a forest. At a time lag of zero, no change in community composition could yet have occurred, and thus the auto-regression of species abundances on themselves has by definition an R^2^ of unity, but as the time elapses between censuses, changes in community composition accumulate and the value of R^2^ decreases.

## Supporting Information

Information S1 (DOC)
