# Supplementary material for: Tropical Forests Are Non-Equilibrium Ecosystems Governed by Interspecific Competition Based on Universal 1/6 Niche Width
Source: PLoS One. 2013 Dec 30;8(12):e82768. doi: 10.1371/journal.pone.0082768 (PMC3875416; doi:10.1371/journal.pone.0082768)
Supplement: Information S1 — (DOC) [file pone.0082768.s001.doc]

**SUPPORTING INFORMATION:**

We used a sequential procedure used to estimate the parameter values provided the best overall fit to the observed metrics that characterised the spatiotemporal community dynamics in the set of censuses of each forest (Fig. 1). Here we illustrate the sensitivity of the RSA to changes in the value of σ estimated for Barro Colorado, the tropical forest for which there is the largest number of censuses (six). We found that, *σ* = 0.077 and *m* = 0.10 provided the best fitting (average *R^2^* = 0.975) for all biodiversity metrics for the six censuses at Barro Colorado. Fig. S1 depicts the observed and RSA distributions for the first census at Barro Colorado for values of *σ* departing from the best estimate of 0.077, showing that the agreement worsened as *σ* became different form its best estimate.


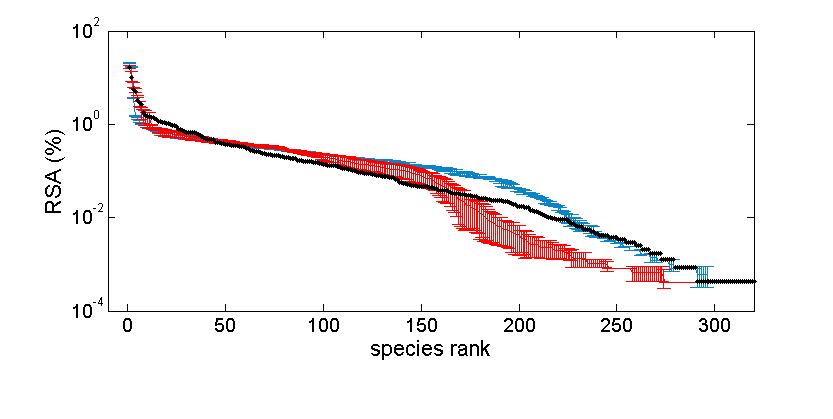


**Fig. S1** Predicted relative abundance distributions agree with observed ones only for a narrow range of niche widths σ and dispersal rates m. The observed relative abundance distribution was calculated from data in [11]. The best parameter estimates for Barro Colorado Island (*σ* = 0.077, *m* = 0.1, *T* = 3) yielded an average coefficient of determination (quantifying the similarity between predicted and observed relative abundance distributions) of 0.975. This agreement between predicted and observed relative abundance distributions worsened for other values of these parameters, being 0.865 for *σ* = 0.06 (blue) and 0.915 when *σ* = 0.1 (red).

Fig. S2 and S3 show the observed and predicted RSA for the consecutive censuses in Barro Colorado and Pasoh. Achieving a good fit for some isolated community metrics in certain censuses of the permanent plots of tropical forests is unlikely to be an impressive feat since this was previously achieved [4,12]. However, we believe that a mechanistic model capable of consistently and accurately predicting an ensemble of community metrics (including some features previously unexplained such as the short-scale temporal aggregation) does represent an important achievement in our understanding of these complex ecosystems.


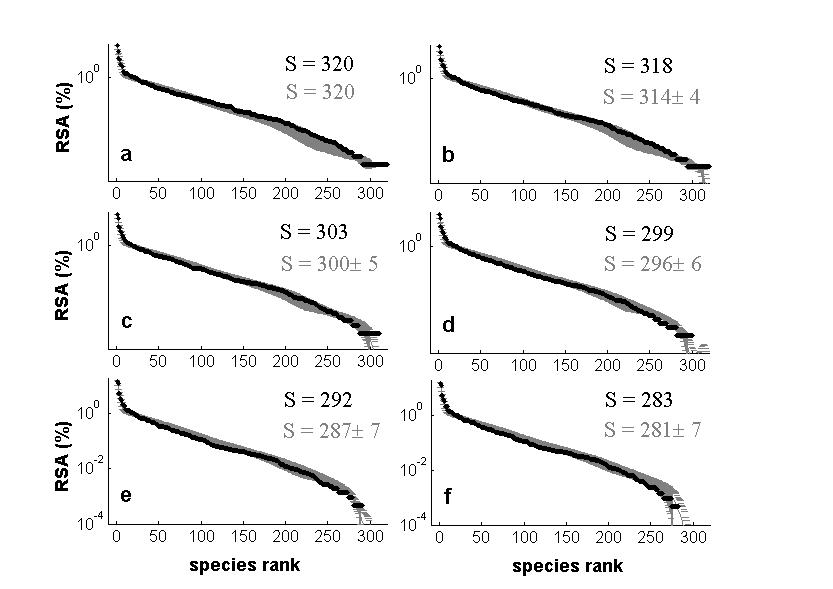


**Fig.S2:** Observed (black) and predicted (gray) species richness and relative species abundance distribution for each complete census of all trees of dbh ≥ 1cm for the six censuses of the Barro Colorado plot. The predicted values of species richness correspond to averages ± std of 100 model simulations for the best estimates of model parameters: *σ* = 0.077, *m* = 0.1, and *T* = 3.. The predicted relative species abundance curves correspond to averages ± std of 100 model simulations for the best estimates of model parameters. **a.** 1982 census. **b.** 1985 census. **c.** 1990 census. **d.** 1995 census. **e.**  2000 census. **f.** 2005 census. (data from [11]).


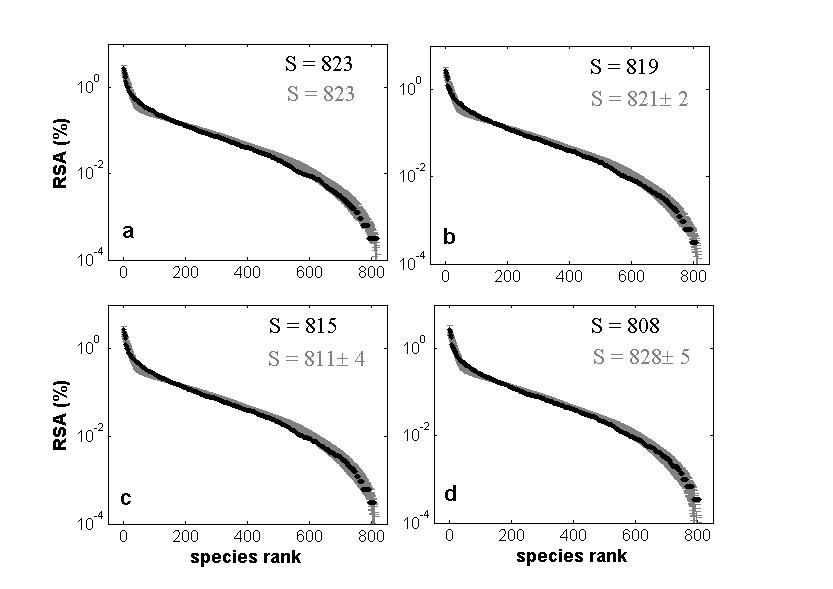


**Fig.S3:** Observed (black) and predicted (gray) species richness and dominance-diversity curves for each complete census of all trees of dbh ≥ 1cm for the four censuses of Pasoh plot. The predicted values of species richness correspond to averages ± std of 100 model simulations for the best estimates of model parameters: *σ* = 0.085, *m* = 0.11, and *T* = 0.5. The predicted relative species abundance curves correspond to averages ± std of 100 model simulations for the best estimates of model parameters. **a.** 1987 census. **b.** 1990 census. **c.** 1995 census. **d.** 2000 census. (Data from [11]).

Finally, it is important to highlight that our model essentially works for saturated forests. To illustrate this limitation we analysed a tenth plot of 50 ha, located in Mudumalai Game Reserve in the Western Ghats of southern India. Mudumalai is an open-canopied forest with a grass-dominated understory and less than 25% total tree cover (Hubbell 2001). The best parameter estimates we could obtain for Mudumalai, *σ* between 0.06 and 0.065; *m* between 0.02 and 0.06; *T* = 0, cannot accurately predict the empirically observed relative abundances of species ranked from 3 to 7 (Fig. S4).


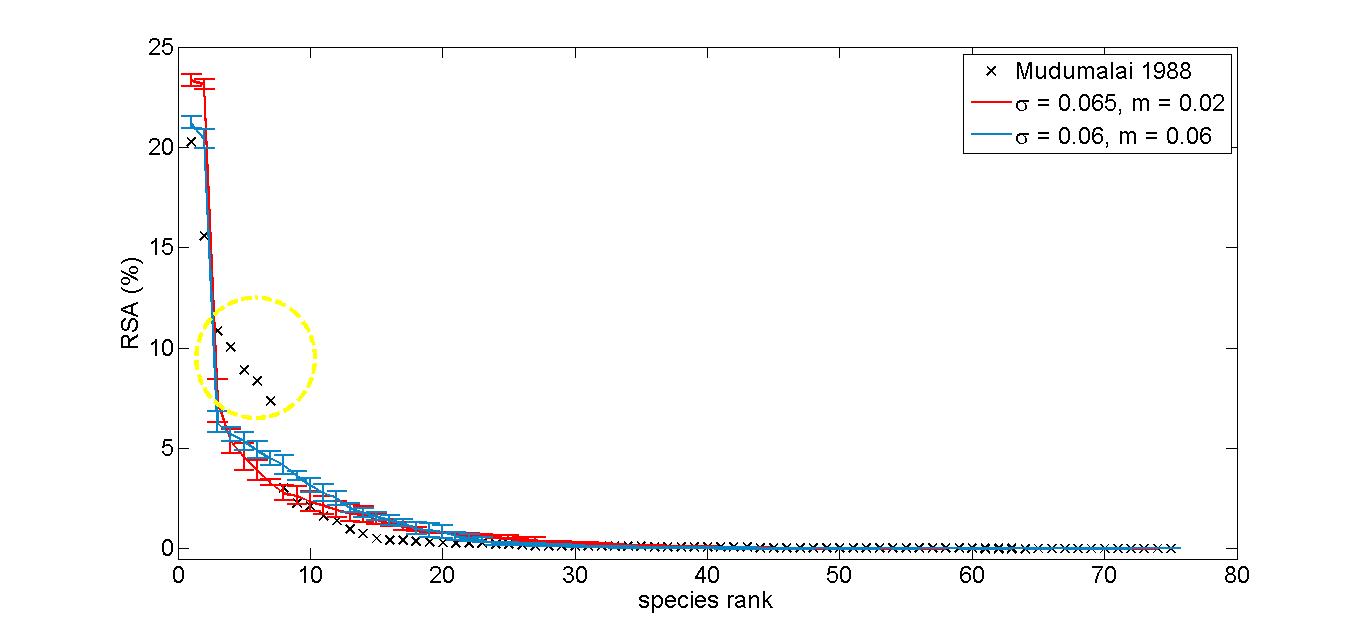


**Fig.S4:** Theoretical vs empirical dominance-diversity curves for Mudumalai showing that theoretical curves fail to reproduce a range of empirically observed abundances (encircled in yellow). The observed relative abundance distribution was calculated from data of [11].
